# Acid production in dental plaque after exposure to probiotic bacteria

**DOI:** 10.1186/1472-6831-12-44

**Published:** 2012-10-24

**Authors:** Mette K Keller, Svante Twetman

**Affiliations:** 1 Department of Odontology, Section of Cariology, Endodontics, Pediatric Dentistry and Clinical Genetics, Faculty of Health Sciences, University of Copenhagen, Copenhagen, Denmark

## Abstract

**Background:**

The increasing interest in probiotic lactobacilli in health maintenance has raised the question of potential risks. One possible side effect could be an increased acidogenicity in dental plaque. The aim of this study was to investigate the effect of probiotic lactobacilli on plaque lactic acid (LA) production *in vitro* and *in vivo*.

**Methods:**

In the first part (A), suspensions of two lactobacilli strains (*L. reuteri* DSM 17938*, L. plantarum* 299v) were added to suspensions of supragingival dental plaque collected from healthy young adults (n=25). LA production after fermentation with either xylitol or fructose was analyzed. In the second part (B), subjects (n=18) were given lozenges with probiotic lactobacilli (*L. reuteri* DSM 17938 and ATCC PTA 5289) or placebo for two weeks in a double-blinded, randomized cross-over trial. The concentration of LA in supragingival plaque samples was determined at baseline and after 2 weeks. Salivary counts of mutans streptococci (MS) and lactobacilli were estimated with chair-side methods.

**Results:**

Plaque suspensions with *L. reuteri* DSM 17938 produced significantly less LA compared with *L. plantarum* 299v or controls (p<0.05). Fructose gave higher LA concentrations than xylitol. In part B, there were no significant differences in LA production between baseline and follow up in any of the groups and no differences between test and placebo were displayed. The salivary MS counts were not significantly altered during the intervention but the lactobacilli counts increased significantly in the test group (p<0.05).

**Conclusion:**

Lactic acid production in suspensions of plaque and probiotic lactobacilli was strain-dependant and the present study provides no evidence of an increase in plaque acidity by the supply of selected probiotic lactobacilli when challenged by fructose or xylitol. The study protocol was approved by The Danish National Committee on Biomedical Research Ethics (protocol no H-2-2010-112).

**Trial registration:**

NCT01700712

## Background

Probiotic bacteria have long been used for improving gastro-intestinal health [1] and there is emerging evidence that lactobacilli-derived probiotic bacteria also can have beneficial influences on oral health. Several studies have shown a decrease in oral mutans streptococci counts after short-term consumption of *L. reuteri*[2-5] and *L. rhamnosus*[6] and a few have demonstrated an effect on caries prevention and control [7-9]. However, less attention has been paid to the potential risk of probiotic usage. Lactobacilli are considered a part of the resident oral microflora but elevated counts have been reported after probiotic regimes [10]. Lactobacilli are highly acidogenic and aciduric but mainly found in the deep carious lesions [11] and hence, not considered to be involved in the initiation of caries lesion. The ability of probiotic lactobacilli to ferment sugars has been shown to be dependent on the type of sugars and to vary between different strains *in vitro*[12]. For example, strains of *L. plantarum* produced acids at a rapid rate while *L. reuteri* slowly generated weak reactions with glucose, lactose, sucrose, maltose and melibiose under both aerobic and anaerobic conditions [12]. However, the clinical relevance of such findings is sparsely investigated. Recently, Marttinen and co-workers [13] reported that intake of tablets containing either *L. rhamnosus* GG or *L. reuteri* did not seem to affect the lactic acid levels in dental plaque. With addition of probiotic bacteria to many commercially available products and their increasing consumption, studies on the safety of lactobacilli intended for oral use are important. Our aim was therefore to investigate the effect of probiotic lactobacilli on lactic acid production in supragingival dental plaque *in vitro* as well as *in vivo*. The null hypothesis was that the acidogenicity would not differ from placebo or controls.

## Methods

The investigation was conducted in two parts; a laboratory study and a double-blinded, randomized cross-over trial. For the clinical part, a power calculation (α=0.05 and β=0.20) based on pilot findings indicated that 18 subjects were needed in the test and placebo groups respectively. The probiotic strains used in both parts are all commercially available and used in fruit drinks and in tablets. The study protocol was approved by The Danish National Committee on Biomedical Research Ethics (protocol no H-2-2010-112)**.** Clinical trial number NCT01700712.

### Part A

Twenty-five healthy young adults of both sexes (mean age 27 yrs) with uncompromised oral health (mean DMFT (Decayed, Missed, Filled Teeth) = 4) were enrolled after informed consent. None of the subjects were habitual consumers of probiotic products. They were instructed to refrain from tooth brushing for 24 hours before their visit. Supragingival dental plaque was collected from all teeth with a blunt explorer, pooled and transferred to a plastic tube. The fresh samples was suspended and homogenized in PBS (Phosphate buffered saline) (pH=7.2) and adjusted to an optical density of OD=0.2 at 340 nm. Five hundred μl of the plaque suspensions were thereafter mixed with 500 μl of equally dense suspensions of either *L. reuteri* DSM 17938 (Biogaia, Stockholm, Sweden) or *L. plantarum* 299v (Probi AB, Lund, Sweden) in PBS. The lactobacilli strains were cultivated in Man Rogosa Sharpe (MRS) broth (Oxoid Ldt., Basingstoke, Hampshire, UK) in an anaerobic incubator at 37°C for 24 h and then washed twice in PBS. One ml of the plaque suspension with no addition of probiotic bacteria served as control. Also, one ml suspensions of lactobacilli with the same optical density as the other samples were used as controls. Baseline spectrophotometric (Genesys 10 uv Scanning, Thermo Scientific, MA, USA) values were recorded and the suspensions were then incubated for 1h in 37°C without agitation. The OD-readings were repeated and the acid production was initiated by adding 25 μl fructose (10%) or xylitol (10%) to each sample. After 30 minutes of further incubation, the fermentation was stopped by centrifugation for 2 min (10,000 rpm) and the supernatant was withdrawn for further analyses. The concentration of the L- and D- isomers of lactic acid (LA) was determined enzymatically and expressed as μg/ml with aid of a commercial kit (EnzyPlus, Biocontrol) according to the manufacturer’s instructions. All assays were performed in duplicate and the mean sum of the L- and D- isomers were calculated.

### Part B

Thirty subjects were screened for the presence of salivary mutans streptococci and eighteen were eligible for the clinical trial (14 female, 4 male; mean age = 26 yr; DMFT= 2). The inclusion criteria were *i)* moderate to high counts of salivary mutans streptococci (>10^4^ CFU) as estimated with the Dentocult SM chair-side test, *ii)* no visible open caries lesions or periodontal disease, and *iii)* being non-smokers. Also, none of the subjects were consuming probiotic products, or had been taking antibiotics within the last two months. After group allocation and a run-in period of 3 days (phase 1) with professional tooth cleaning, the participants were instructed to take three tablets per day (morning, noon and evening) containing either two strains of the probiotic bacterium *L. reuteri* (DSM 17938 and ATCC PTA 5289; 1x10^8^ CFU of each strain) or placebo for 2 weeks (phase 2). Both tablets were provided by BioGaia AB (Sweden). Stimulated saliva samples were collected after 3 minutes of chewing paraffin gums. Pooled supragingival plaque samples were collected at baseline and after the intervention. Numbers of mutans streptococci and *Lactobacilli* in saliva was determined by the aid of a test kit (Dentocult SM Strip Mutans and Dentocult LB respectively, Orion Diagnostica, Finland) according to the manufacturer’s guidelines. The samples were all collected between 10 and 11 am before lunch and approximately 3–4 hours after consuming the last tablet. After a 3-week washout period (phase 3), the 2-week intervention was repeated with the second tablet (phase 4). Again, baseline and follow-up samples were collected. The tablets were supplied and coded A or B by the manufacturer and the test and placebo products were identical in taste, composition and appearance. The code was not unveiled until after the analyses and the statistical calculations. During all phases, the subjects were asked to maintain their normal oral hygiene routines including tooth brushing with fluoride toothpaste. They were however firmly requested to avoid intake of any food items supplemented with probiotic bacteria.

### Laboratory assays

All analysis and bacterial counts were made without knowledge of group allocation. The salivary samples were incubated in 2 and 4 days respectively at 37°C for the presence of mutans streptococci and *lactobacilli* with aid of selective chair-side tests (Dentocult SM and LB, respectively; Orion Diagnostica, Helsinki, Finland) according to the manufacturer’s instructions. The colony forming units were counted in a stereomicroscope (12–25 x magnification) by one single examiner and scored in categories as described in Tables 1 and 2. The concentration of LA in the plaque samples was determined in duplicate as described above using the fresh plaque samples.

**Table 1:** Salivary lactobacilli score

	**Lactobacilli score**				
	**0 (0 CFU/ml)**	**1 (10**^3 ^**CFU/ml)**	**2 (10**^4 ^**CFU/ml)**	**3(>10**^5 ^**CFU/ml)**	**p**
Test BL	9	4	1	4	< 0.05
Test FU	0	4	10	4	
Placebo BL	7	5	4	2	NS
Placebo FU	5	6	4	3	

Samples collected from subjects (n=18) before (BL) and after (FU) a 2-week intervention period with *L. reuteri* or placebo. NS = not significant.

**Table 2:** Mutans streptococci score

	**Streptococcus mutans score**				
	**0 (<10**^3 ^**CFU/ml)**	**1 (10**^3^**<10**^4 ^**CFU/ml)**	**2 (10**^4^**<10**^5 ^**CFU/ml)**	**3 (≥10**^5 ^**CFU/ml)**	**p**
Test BL	2	4	8	4	NS
Test FU	1	7	6	4	
Placebo BL	2	5	6	5	NS
Placebo FU	1	4	9	4	

Samples collected from subjects (n=18) before (BL) and after (FU) a 2-week intervention period with *L. reuteri* or placebo. NS = not significant.

### Statistical methods

Data was processed with the IBM-SPSS software (version 19.0, Chicago Ill, USA). In part A, differences in outcome between probiotic strains vs. control was analyzed with Wilcoxon sign rank test. In part B, acid production was compared before and after intervention within groups as well as between the groups before and after intervention by Wilcoxon signed rank test. Differences in lactobacilli and mutans streptococci were analyzed by Chi square test. A p value <0.05 was considered statistically significant. Reliability of the bacterial scoring was analyzed on a random sample (20%) of the tests and the intra examiner correlation showed an ICC value of 0.97.

## Results

### Part A

**Figure 1:**
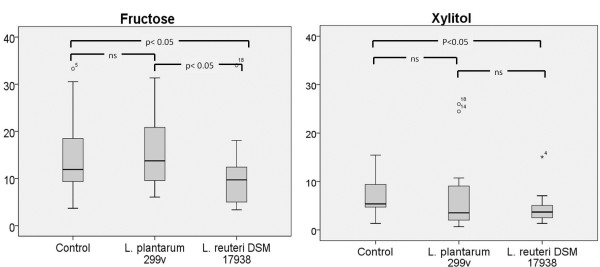
**Mean lactic acid production in plaque suspensions and with addition of *L. plantarum *299v *and L. reuteri *DSM 17938 after fermentation of fructose (a) and xylitol (b).** The controls are plaque suspensions without probiotic bacteria. The values shown are μg/mL after 30 min fermentation. ns = not significant.

All plaque samples from the 25 subjects produced detectable levels of lactic acid and the levels initiated from fructose were significantly higher than those from xylitol (Figure 1). However, considerable inter-individual variations were noted. After fermentation with fructose, the plaque suspensions mixed with *L. reuteri* DSM 17938 produced significantly less LA compared with both the control samples and the samples with *L. plantarum* 299v (p<0.05). There were no statistical significant differences between the control samples and the samples with *L. plantarum* 299v. The control suspensions containing only one of the two lactobacilli both showed acid production of the same magnitude as the plaque control (*L. reuteri* DSM 17938: mean 6.0 (4.8) and *L. plantarum* 299v: mean 9.3 (5.9)). After acid production initiated with xylitol, a statistically significant (p<0.035) difference between the control sample and the samples with *L. reuteri* DSM 17938 was obtained. However, no significant differences between the control samples and the samples with *L. plantarum* 299v or between the two samples with added probiotic lactobacilli were seen. The samples where xylitol was added to pure suspensions of either of the lactobacilli both showed slightly lower values than the plaque suspension, but did not differ significantly (*L. reuteri* DSM 17938: mean 0.5 ( 3.4) and *L. plantarum* 299v: mean 3.4 (3.5)).

### Part B

There were no major differences in LA concentration between the samples collected at baseline and follow up in either the test or the placebo group (Table 3). Again, the samples supplied with fructose as a substrate displayed significantly higher levels of produced lactic acid than those with added xylitol. The salivary lactobacilli scores in the test group increased significantly (p<0.05) from baseline to follow-up but no differences were seen in the placebo group (Table 1). At baseline, nine participants in the test group presented non-detectable levels of lactobacilli versus six in the placebo group. After two weeks, all subjects in the test group displayed detectable counts while five subjects in the placebo group still had levels beyond detection. In Table 2, the salivary mutans scores are presented. No statistically significant effects were observed during the experimental periods in any of the groups. No side effects following the use of the probiotic lozenges were reported.

**Table 3:** Mean lactic acid concentration (SD) μg/ml in suspensions of pooled supragingival plaque from health young adults (n=18)

**Group**	**BL**		**FU**	
	**Fructose**	**Xylitol**	**Fructose**	**Xylitol**
Test	8.6 (5.4)	2.9 (2.0)	8.7 (4.7)	1.5 (3.5)
Placebo	9.5 (4.2)	3.2 (3.5)	10.8 (5.2)	2,9 (3.4)

Measurements made at baseline (BL) and follow-up (FU) after the two week intervention period with *L. reuteri* (test) or placebo. Acid production was initiated with either fructose or xylitol.

## Discussion

The increased interest in using of probiotic lactobacilli to improve oral health has raised concerns on its possible side effects. One side effect from a cariological point of view would be an increased production of organic acids in the dental plaque. Therefore, we wanted to investigate if addition of probiotic lactobacilli to dental plaque would influence its acidogenicity. In the laboratory part, we found clear differences between the lactic acid production in suspensions of pooled plaque and *L. reuteri* DSM 17938 on one hand and plaque plus *L. plantarum* 299v or pure controls on the other. This was in agreement with the *in vitro* study of Hedberg et al. [12], who found *L. plantarum* 299v to be most prone to produce acid among six different commercial strains tested. Conversely, Haukioja and co-workers [14] found that both *L. reuteri* DSM (formerly ATCC 55730) and *L. plantarum* 299v lowered the pH significantly after fermentation of glucose and sucrose. According to Hedberg et al. [12], the fermentation of sucrose and glucose by *L. reuteri* PTA 5289 resulted in a lowering of the pH (pH 5.2-6.8), whereas the fermentation of fructose caused a minor increase in pH (pH>6.8). This, together with our present results, indicates that the sugar chosen for fermentation has a strong impact on the LA production of *L. reuteri*. Thus, from the first part of this study, it seems clear that the acidogenicity of suspensions of dental plaque and probiotic bacteria is strain-dependant and highly influenced by the type of sugar available. Moreover, it was noteworthy that the results of part A were completely in line with findings of previous *in vitro* studies [12,14]. This indicates that the laboratory methods for screening probiotic candidates for oral diseases may be useful to differentiate between wanted and unwanted targeted properties. As acid production in suspensions in the laboratory may not mimic the exact conditions in of the complex biofilm *in situ*, a step further could be to study the pH of the plaque *in vivo* by a micro pH-meter.

In the clinical part of this study we found no differences in acid production before and after the intervention with *L. reuteri* or placebo when the plaque samples were fermenting fructose. Thus, the null hypothesis could not be rejected. The findings were in accordance with Marttinen et al. [13], who failed to demonstrate any differences in lactic acid production after the intervention with either *L. reuteri* or *L. rhamnosus* GG. It could be argued that the decreased acidogenity of the plaque samples could be caused by the antibacterial activity of L. reuteri, but due to the relatively short period of administration we believe that this was not the case. Since xylitol is not thought to be fermented by oral bacteria [15] the low concentrations of LA after addition of xylitol were expected. It should however be stressed that the volunteers in this projects had healthy oral conditions and most likely not fully representative for subjects with an ecologically stressed and unbalanced biofilm. In theory, such a condition could possibly increase the efficacy of probiotic therapy. We and other research groups have demonstrated that low pH conditions may promote mutans streptococcus growth inhibition by probiotic lactobacilli [16,17] and interfere with the biofilm formation [18]. Thus, further studies on selected caries-active patients would be interesting. The conclusion so far from the clinical part was that despite their strong acidogenic abilities, daily supplements of probiotic lactobacilli did not seem to increase the acidogenity of the dental plaque when exposed to fructose or xylitol.

There were no differences in salivary mutans streptococci levels between the two groups and we recorded no changes after the intervention period. This was in contrast to several previous studies [2-5] but in concert with others [9,19,20]. Differences in strains and bacterial concentrations, dose regimes and age groups used in the earlier trials hamper any comparison between the trials and thereby firm conclusions. It is generally believed that probiotic supplements may be more effective in preschool ages than in adults [21], which to some extent may explain our negative findings. The increased in lactobacilli counts in the test group was logical and supported the findings of Marttinen and co-workers [13] although we were not able to analyze whether or not it was due to the specific strain tested. However, it is unlikely that a permanent shift of the microbial composition occurs [22,23]. Caglar et al. [22] studied the number of carriers of *L. reuteri* ATCC 55730 after a 2-week intake period of tablets containing that strain. Using selective media and analyzes for reuterin production, the bacteria was recovered in all test subjects immediately after the intervention period but it was gradually eliminated within a few weeks. However, it is still an open question if a colonization of the oral biofilm *per se* is an absolute requirement for probiotic action.

## Conclusion

The results of the present study suggest that the acidogenicity of suspension of dental plaque and probiotic lactobacilli is strain-dependant and influenced by the type of sugar available. Furthermore, The present study provides no evidence of an increase in plaque acidity by the supply of selected probiotic lactobacilli when challenged by fructose or xylitol.

## Competing interests

MKK have received a research grant from Biogaia AB, Sweden for clinical trials on oral probiotics.

## Authors’ contributions

MKK contributed with the laboratory work, the acquisition, analysis and interpretation of data and drafting of the manuscript. ST contributed with the design, interpretation of data and revision of the manuscript. All authors read and approved the final manuscript.

## Pre-publication history

The pre-publication history for this paper can be accessed here:

http://www.biomedcentral.com/1472-6831/12/44/prepub
